# Author Correction: Phase separation of initiation hubs on cargo is a trigger switch for selective autophagy

**DOI:** 10.1038/s41556-026-02095-4

**Published:** 2026-09-23

**Authors:** Mariya Licheva, Jeremy Pflaum, Riccardo Babic, Hector Mancilla, Jana Elsässer, Emily Boyle, David M. Hollenstein, Jorge Jimenez-Niebla, Jonas Pleyer, Mio Heinrich, Franz-Georg Wieland, Joachim Brenneisen, Christopher Eickhorst, Johann Brenner, Shan Jiang, Markus Hartl, Sonja Welsch, Carola Hunte, Jens Timmer, Florian Wilfling, Claudine Kraft

**Affiliations:** 1 Institute of Biochemistry and Molecular Biology, ZBMZ, Faculty of Medicine, University of Freiburg, Freiburg, Germany; 2 Faculty of Biology, University of Freiburg, Freiburg, Germany; 3 Mechanisms of Cellular Quality Control, Max Planck Institute of Biophysics, Frankfurt am Main, Germany; 4 Spemann Graduate School of Biology and Medicine (SGBM), University of Freiburg, Freiburg, Germany; 5 Department for Biochemistry and Cell Biology, University of Vienna, Center for Molecular Biology, Vienna Biocenter Campus (VBC), Vienna, Austria; 6 Mass Spectrometry Facility, Max Perutz Labs, Vienna Biocenter Campus (VBC), Vienna, Austria; 7 Freiburg Center for Data Analysis and Modelling (FDM), University of Freiburg, Freiburg, Germany; 8 CIBSS - Centre for Integrative Biological Signalling Studies, University of Freiburg, Freiburg, Germany; 9 Institute of Physics, University of Freiburg, Freiburg, Germany; 10 Central Electron Microscopy Facility, Max Planck Institute of Biophysics, Frankfurt am Main, Germany; 11 BIOSS-Centre for Biological Signalling Studies, University of Freiburg, Freiburg, Germany

**Keywords:** Autophagy, Organelles

Correction to: *Nature Cell Biology* https://doi.org/10.1038/s41556-024-01572-y, published online 7 January 2025.

In the version of the article initially published, a copy–paste error during data preparation resulted in incorrect data being used in Extended Data Fig. 6c and its source data. The figure has been regenerated using the correct original data, and the Source Data file has been updated accordingly. During a subsequent review of the Source Data for all other figures, an additional copy–paste error was identified in the Extended Data Fig. 5a source data. The data used to generate Extended Data Fig. 5a were correct, and the figure itself is therefore unaffected. The Source Data file for Extended Data Fig. 5a has also been corrected and the amended file is now available online. These corrections do not affect the interpretation, results or conclusions of the article.

