# Supplementary material for: Phase separation of initiation hubs on cargo is a trigger switch for selective autophagy
Source: Nat Cell Biol. 2025 Jan 7;27(2):283–97. doi: 10.1038/s41556-024-01572-y (PMC11821514; doi:10.1038/s41556-024-01572-y)
Supplement: Supplementary file 1 — Supplementary Note 1. [file 41556_2024_1572_MOESM1_ESM.pdf]

nature cell biology

Supplementary information https://doi.org/10.1038/s41556-024-01572-y

Phase separation of initiation hubs on cargo is a trigger switch for selective autophagy

In the format provided by the authors and unedited

Supplementary Note 1:

Development of mathematical model of Atg11/Atg19-cargo interaction

We developed a mathematical model to elucidate the difference between avidity-driven interactions and affinity-driven interactions for the assembly of initiation hubs. In the following, the mathematical foundations and assumptions of the model and the implementation in simulations are introduced. For this purpose, first, the mechanics of the individual agents of the agent-based model and their interactions are defined. Subsequently, the numerical implementation of the simulations is introduced and finally, the results of the modeling show that simulations based on low-affinity but high-avidity-mediated interactions produced a pattern of Atg11/Atg19 clusters on the cargo, while high-affinity interactions could not produce these clusters.

Mechanics of agent-based models

The developed model consists of agents which represent the proteins of interest and constituents of the cargo. As Atg11 and Atg19 interact with very high affinity, they were assumed as one entity for simplification (Atg11w19). We model the positions of all agents by soft spheres. Their interactions are given by forces resulting from an interaction potential. To describe the stochastic molecular motion together with forces arising due to interactions, we use Brownian dynamics given by

$$\frac{\partial X}{\partial t} = -\frac{D}{k_B T}\nabla V(X) + \sqrt{2D}R(t) \quad (1)$$

where $R(t)$ is the increment of the Wiener process which produces a gaussian white noise term in the motion of the particles. The full list of all parameters can be found in Table M1.

Affinity-interactions of particles based on force-potentials

Particles interact via a predefined force-potential which can be adapted by modifying its parameters. The force can be calculated by the following equations. The variable r indicates the distance between the two particles i and j of interest while r_i and r_j describe their radii respectively. The non-dimensional variable σ is just used to simplify some of the equations. A value of $\sigma < 1$ means that the two interacting soft spheres are overlapping and the resulting force should be repelling the two. Conversely, $\sigma > 1$ means that the interacting particles are separated and do not overlap and attract each other.

$$\sigma(r) = \frac{r}{r_i + r_j} \quad (2)$$

The strength of the interaction can then be calculated via

$$P(r) = \frac{1}{\sigma(r)^4} - \frac{1}{\sigma(r)^2}. \quad (3)$$

We can see that the overall sign is positive when $\sigma < 1$ and negative in the other case. This leads to repelling and attracting forces. For numerical stability, we have to enforce an upper bound β on the interaction strength. We see that during the simulation particles very rarely reach this bound but it is a safety mechanism to keep the simulation running in the rare case that this happens. The value 4 can be calculated by considering two particles which with the combined radial value $r = (r_i + r_j)/\sqrt{2}$. This is equivalend to $\approx 0{,}29\%$ overlap. The quartic term in the potential strength $P(r)$ produces the value 4 which is the basis of the chosen

bound. To not neglect the changing strength of the individual interactions completely, we introduce an additional term of $1/\sigma(r)$.

$$\beta = 4 + \frac{1}{\sigma(r)} \tag{4}$$

Furthermore, it is often necessary to restrict the interaction range of such potentials. We introduce the interaction range ζ. Since we assume that our particles are not point-like but rather extend in space as soft spheres, we need to add the radius of the current particle and the interacting particle to the total interaction range to obtain the actual cutoff of the potential.

$$c = \zeta + r_i + r_j \tag{5}$$

The complete interaction potential can then be calculated via

$$V(r) = U_{i,j} min(P(r), \beta)\theta(c - r) \tag{6}$$

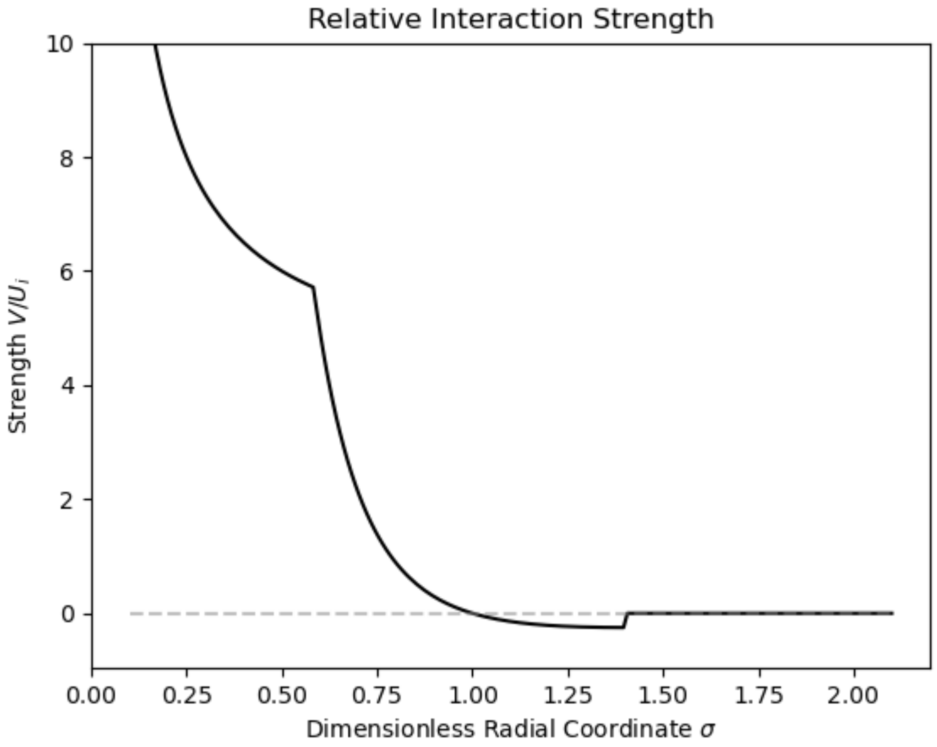

where θ is the Heaviside step-function which enforces the interaction cutoff given by β and $U_{i,j}$ is the interaction strength between particle i and partile j.

Figure M1: Shape of the force acting between particles. The cutoff was chosen to be $c = 1.5\sigma$. Since the plot shows the relative interaction strength, this is the only parameter.

Parameter	Description
N_c, N_a	Number of particles in the simulation
$k_B T$	Thermodynamic energy
D	Diffusion Constant
r_c, r_a	Radii of the particles
$U_{c,c}, U_{c,a}, U_{a,a}$	Interaction strengths between particle species
$\zeta_{c,c}, \zeta_{c,a}, \zeta_{a,a}$	Interaction ranges

Table M1: Parameters of the simulation. Variables with index c correspond to properties of the cargo particles while a corresponds to Atg11w19.

Avidity and Affinity Interactions
To model the avidity case, which is the cooperation of neighboring molecules in attracting another species of particles, we find adjust the potential strength between Atg11w19 and the Cargo constituent particles such that the final simulation result yields a clustered pattern on the surface of the Cargo. All remaining parameters remain fixed. The result of the three presented cases for high Cargo-Atg11w19 interaction strengths is displayed in the main text.

Numerical Implementation
We used `cellular_raza` (https://github.com/jonaspleyer/cellular_raza) to solve the equations of motion.

Initial Positions
Cargo
Initially, Cargo particles are placed randomly inside a sphere with a given radius. They are then propagated until they reach an equilibrium state. This serves as the initial state for the next simulation step.
Atg11w19
After the cargo particles have reached equilibrium, we introduce the Atg11w19 particles into the simulation. They are placed randomly outside of a sphere which radius is given by the initial cargo placements and an additional band which acts as a spacer.

Simulation Flow
For the second simulation step in which Cargo and Atg11w19 particles are combined, the position of cargo particles is fixed in space for the remainder of the simulation time. Meanwhile, the Atg11w19 particles are subject to the motions described above. If a particle collides with the simulation boundary, it is reflected. However, this behavior only occurs infrequently in our simulation.

Storing Results
One particle can be represented as a collection of all its parameters. They are stored in multiple files. A typical section of such a file is represented in Figure M2. Similarly, the overall settings required to run a single simulation are also stored in a single file. This is automatically done by cellular_raza in the background.

```
{
  "mechanics": {
    "pos": [
    8.857474173057744e-7,
    1.6095030291306556e-6,
    9.080380540878439e-7
    ],
    "diffusion_constant": 4.8000000000000003e-17,
    "kb_temperature": 4.141947e-21,
    "random_vector": [
    -0.7010850624988277,
    5.667906385578381,
    -3.523344099774444
    ]
  },
  "interaction": {
    "species": "Atg11w19",
    "cell_radius": 1.0000000000000001e-7,
    "potential_strength_cargo_cargo": 3e-12,
    "potential_strength_atg11w19_atg11w19": 6.2e-13,
    "potential_strength_cargo_atg11w19": 1e-12,
    "interaction_range_cargo_cargo": 8.000000000000001e-8,
    "interaction_range_atg11w19_atg11w19": 8.000000000000001e-8,
    "interaction_range_atg11w19_cargo": 1.2000000000000002e-7,
    "relative_neighbour_distance": 1.5,
    "neighbour_count": 0
  }
}
```

Figure M2: Json representation of a single particle of species Atg11w19.

```
{
  "n_cells_cargo": 220,
  "n_cells_atg11w19": 360,
  "cell_radius_cargo": 1.0000000000000001e-7,
  "cell_radius_atg11w19": 1.0000000000000001e-7,
  "diffusion_cargo": 5e-17,
  "diffusion_atg11w19": 4.8000000000000003e-17,
  "temperature_atg11w19": 300.0,
  "temperature_cargo": 300.0,
  "potential_strength_cargo_cargo": 3e-12,
  "potential_strength_cargo_atg11w19": 5e-13,
  "potential_strength_atg11w19_atg11w19": 6.2e-13,
  "interaction_range_cargo_cargo": 8.000000000000001e-8,
  "interaction_range_atg11w19_cargo": 1.2000000000000002e-7,
  "interaction_range_atg11w19_atg11w19": 8.000000000000001e-8,
  "relative_neighbour_distance": 1.5,
  "dt": 0.12,
  "t_max": 8400.0,
  "save_interval": 6.0,
  "extra_saves": [],
  "n_threads": 4,
  "domain_size": 5e-6,
  "domain_cargo_radius_max": 6.000000000000001e-7,
  "domain_atg11w19_radius_min": 6.5e-7,
  "domain_n_voxels": 5,
  "storage_name": "out/autophagy_param_space",
  "substitute_date": "0000000601",
  "cargo_initials_dir": "out/cargo_initials",
  "show_progressbar": false,
  "random_seed": 4
}
```
Figure M3: All settings needed to specify a complete simulation run.
