# Supplementary material for: Phase separation of initiation hubs on cargo is a trigger switch for selective autophagy
Source: Nat Cell Biol. 2025 Jan 7;27(2):283–97. doi: 10.1038/s41556-024-01572-y (PMC11821514; doi:10.1038/s41556-024-01572-y)
Supplement: Supplementary file 2 — Reporting Summary [file 41556_2024_1572_MOESM2_ESM.pdf]

nature portfolio

Corresponding author(s): Claudine Kraft
Florian Wilfling

Last updated by author(s): 26/10/2024

Reporting Summary

Nature Portfolio wishes to improve the reproducibility of the work that we publish. This form provides structure for consistency and transparency in reporting. For further information on Nature Portfolio policies, see our Editorial Policies and the Editorial Policy Checklist.

Statistics

For all statistical analyses, confirm that the following items are present in the figure legend, table legend, main text, or Methods section.

n/a	Confirmed	
☐	☒	The exact sample size (*n*) for each experimental group/condition, given as a discrete number and unit of measurement
☐	☒	A statement on whether measurements were taken from distinct samples or whether the same sample was measured repeatedly
☐	☒	The statistical test(s) used AND whether they are one- or two-sided<br>*Only common tests should be described solely by name; describe more complex techniques in the Methods section.*
☒	☐	A description of all covariates tested
☒	☐	A description of any assumptions or corrections, such as tests of normality and adjustment for multiple comparisons
☐	☒	A full description of the statistical parameters including central tendency (e.g. means) or other basic estimates (e.g. regression coefficient) AND variation (e.g. standard deviation) or associated estimates of uncertainty (e.g. confidence intervals)
☐	☒	For null hypothesis testing, the test statistic (e.g. *F*, *t*, *r*) with confidence intervals, effect sizes, degrees of freedom and *P* value noted<br>*Give P values as exact values whenever suitable.*
☒	☐	For Bayesian analysis, information on the choice of priors and Markov chain Monte Carlo settings
☒	☐	For hierarchical and complex designs, identification of the appropriate level for tests and full reporting of outcomes
☒	☐	Estimates of effect sizes (e.g. Cohen's *d*, Pearson's *r*), indicating how they were calculated

Our web collection on statistics for biologists contains articles on many of the points above.

Software and code

Policy information about availability of computer code

Data collection	CryoEM: LAS X Navigator software (Leica Microsystems, version 3.5.7.23225); SerialEM, AutoTEM software (Thermo Scientific, version 2.4), 3D-Correlation Toolbox (https://3dct.semper.space/)<br>Fluorescence microscopy: Fluorescence microscopy images were captured using the DeltaVision OMX Flex microscope with AcquireSR software (version 4.5.10170-1), the DeltaVision Ultra High Resolution microscope with AcquireUltra software (version 1.2.2), the Leica Stellaris 5 system with Leica Application Suite X (version 4.6.,1.27508) software, or the Nikon Eclipse Ti2 microscope with NIS-Elements AR 5.10.00 software. Raw microscopy images acquired with the DeltaVision Ultra High Resolution microscope or DeltaVision OMX Flex microscope were deconvolved using the softWorX deconvolution plugin (version 7.2.1 and version 7.2.0, respectively). Raw microscopy images acquired with the Nikon Eclipse Ti2 microscope were deconvolved using the NIS Elements Batch Deconvolution v5.20.00. Chromatic shift correction was done with Huygens compute engine 23.04.0p0.<br>Flow cytometry: CytExpert 2.3 analysis software
Data analysis	CryoEM: Huygens Essential version 23.04.0p0, MAPS Software (Thermo Scientific), AreTomo version 1.3.3; cryoCARE; MemBrain-Seg; Amira (Thermo Scientific, version 2022.2), https://github.com/juglab/cryoCARE_pip, https://github.com/teamtomo/membrain-seg<br>Fluorescence microscopy: FIJI, SoftWorX (version 7.2.1 and version 7.2.0, respectively), NIS Elements Batch Deconvolution v5.20.00<br><br>Flow cytometry: FlowJo (FlowJo V.10.9.0 - May 5, 2023), in-house developed Python library mKeima (version 0.5.0, available at https://pypi.org/project/mkeima); Imaris Image Analysis Software Version 10.2 (Oxford Instruments Andor), https://github.com/hollenstein/sourcecode_mkeima-assay_licheva-et-al-2024<br><br>Mass spec: HTRMS converter (version 18.3, Biognosys), Spectronaut (version 18.5, Biognosys), the in-house developed Python library

MsReport (version 0.0.23), ModeNormalizer from MsReport; Linear Models for Microarray Analysis (LIMMA, version 3.54.2) package in R.

Mathematical modeling: cellular_raza (https://github.com/jonaspleyer/cellular_raza)

FRAP:https://github.com/CraignRush/FRAP-Processing

For manuscripts utilizing custom algorithms or software that are central to the research but not yet described in published literature, software must be made available to editors and reviewers. We strongly encourage code deposition in a community repository (e.g. GitHub). See the Nature Portfolio guidelines for submitting code & software for further information.

Data

Policy information about availability of data

All manuscripts must include a data availability statement. This statement should provide the following information, where applicable:

- Accession codes, unique identifiers, or web links for publicly available datasets
- A description of any restrictions on data availability
- For clinical datasets or third party data, please ensure that the statement adheres to our policy

We believe in making data publicly available whenever possible and do so for most of our data. The microscopy data is derived from hypothesis-driven experiments, unlike, for example, screening data, and mining it will be of limited use. However, as these images take up a lot of storage space and are cumbersome to deposit, we feel it's better to make the data available on request to those who need it.

No restrictions apply to the data collected for this manuscript. The mass spectrometry proteomics data have been deposited to the ProteomeXchange Consortium via the PRIDE 55 partner repository with the data set identifier PXD047277. Source data have been provided in Source Data. All other data supporting the findings of this study are available from the corresponding author on reasonable request.
The Python source data are available from the GitHub repository at https://github.com/hollenstein/sourcecode_mkeima-assay_licheva-et-al-2024
Datasets used: PDB: 3OGO, gene reference proteome from Uniprot (Proteome ID: UP000005640, release 2023.03), concatenated with a database of 379 common laboratory contaminants (in-house database), Gene Ontology (GO) term “ER membrane” (GO:0005789)

Research involving human participants, their data, or biological material

Policy information about studies with human participants or human data. See also policy information about sex, gender (identity/presentation), and sexual orientation and race, ethnicity and racism.

Reporting on sex and gender	n.a.
Reporting on race, ethnicity, or other socially relevant groupings	n.a.
Population characteristics	n.a.
Recruitment	n.a.
Ethics oversight	n.a.

Note that full information on the approval of the study protocol must also be provided in the manuscript.

Field-specific reporting

Please select the one below that is the best fit for your research. If you are not sure, read the appropriate sections before making your selection.

☒ Life sciences ☐ Behavioural & social sciences ☐ Ecological, evolutionary & environmental sciences

For a reference copy of the document with all sections, see nature.com/documents/nr-reporting-summary-flat.pdf

Life sciences study design

All studies must disclose on these points even when the disclosure is negative.

Sample size	No statistical methods were used to predetermine sample size. A sufficient sample size was determined based on variance between experiments.
Data exclusions	No data was excluded from analysis, except for clear technical failure.
Replication	At least three successful independent biological replicates were performed for each experiment, as indicated in the material and method section, except two successful biological replicates for Figure S7b, and three technical replicates for Fig. 1e,f and ED Fig. 3a,f,g, 4c,d.
Randomization	For all fluorescence microscopy image acquisition, cells were selected at random from the brightfield channel without bias towards the fluorescence signal. None microscopy samples (e.g. immunoblots) were analyzed based on genotypes, treatments, and/or time points with

internal controls without randomization.

Blinding

For some experiments, blinding of the manual data analysis was not performed as all cells in the images were analyzed and field of views were selected from the brightfield without prior knowledge of the fluorescence signal (Fig. 1d, 2f; ExtData 3e, 5b).

For all other fluorescence microscopy experiments cells were selected at random from the brightfield channel without bias towards the fluorescence signal. Analysis of fluorescence images was either performed computationally (no blinding necessary) (Fig. 1b, 1c, 3a, 3g; ExtData 1c, 1g, 1h) or when manual performed quantification of fluorescence microscopy images was performed blindly after randomizing file names (all others).

Reporting for specific materials, systems and methods

We require information from authors about some types of materials, experimental systems and methods used in many studies. Here, indicate whether each material, system or method listed is relevant to your study. If you are not sure if a list item applies to your research, read the appropriate section before selecting a response.

Materials & experimental systems

n/a	Involved in the study
☐	☒ Antibodies
☐	☒ Eukaryotic cell lines
☒	☐ Palaeontology and archaeology
☒	☐ Animals and other organisms
☒	☐ Clinical data
☒	☐ Dual use research of concern
☒	☐ Plants

Methods

n/a	Involved in the study
☒	☐ ChIP-seq
☐	☒ Flow cytometry
☒	☐ MRI-based neuroimaging

Antibodies

Antibodies used

mouse monoclonal anti-GFP (1:100; 2B6, Monoclonal Antibody Facility, Max Perutz Labs, Vienna)
mouse monoclonal anti-GFP (1:5,000; 7.1 and 13.1, Ref No. 11814460001, Lot No. 70378300, Roche)
IRDye 800CW Goat anti-Mouse (1:1,000, Ref No. 926-32210, Lot No. D10825-15, Licor),
anti-Pgk1 (1:10,000, 22C5D8, Ref No. 459250 Lot NoVC2958788, Invitrogen)

rabbit polyclonal anti-Ape1 (1:15,000), was generated by immunizing rabbits with a synthetic peptide corresponding to amino acids 168-182. ref 43.

rabbit polyclonal anti-Atg19 (1:5,000, Sascha Martens, Monoclonal Antibody Facility, Max Perutz Labs, Vienna) ref 9

mouse monoclonal anti-GST (1:1,000, 2H3-D10, Monoclonal Antibody Facility, Max Perutz Labs, Vienna)

rabbit polyclonal anti-ATG13, (1:50, 5HY-C1-F8, Monoclonal Antibody Facility, Max Perutz Labs, Vienna) ref 44

mouse monoclonal anti-RFP (1:1,000, 6g6, Ref No. 6g6-100 Lot No51020014AB-05, Chromotek). validated by the company using transient expression of mRFP, mCherry, mPlum, mOrange, mRFPruby, DsRed, mScarlet and tdTomato on HEK 293T cells by western blot. Ref: Barucci G et al., Nat Cell Biol. 2020, doi:10.1038/s41556-020-0462-7.

Validation

mouse monoclonal anti-GFP (1:100; 2B6, Monoclonal Antibody Facility, Max Perutz Labs, Vienna) validated by the facility by western blot (GFP transfected vs. untransfected cells), used in Kiermaier et al., Science 2016, doi: 10.1126/science.aad0512

mouse monoclonal anti-GFP (1:5,000; 7.1 and 13.1, Ref No. 11814460001, Lot No. 70378300, Roche), The mixture of two monoclonal antibodies, clones 7.1 and 13.1 was validated and tested by the company using western blot and immunoprecipitation of GFP fusion proteins. Ref: Wong et al., Blood. 2011, doi:10.1182/blood-2011-06-353938.

IRDye 800CW Goat anti-Mouse (1:1,000, Ref No. 926-32210, Lot No. D10825-15, Licor), Validated and tested by the company using dot blot and solid phase absorbed for minimal cross-reactivity with human, rabbit, goat, rat, and horse serum proteins. Ref: Wallroth et al., Nat Cell Biol 2019, doi:10.1038/s41556-019-0377-3.

anti-Pgk1 (1:10,000, 22C5D8, Ref No. 459250 Lot NoVC2958788, Invitrogen), validated by the company using Saccharomyces cerevisiae cell lysate and western blot. Ref: Montellà-Manuel et al., Int J Mol Sci. 2023, doi:10.3390/ijms24032438.

rabbit polyclonal anti-Ape1 (1:15,000), described and validated for yeast by western blot in ref 43

rabbit polyclonal anti-Atg19 (1:5,000, Sascha Martens, Monoclonal Antibody Facility, Max Perutz Labs, Vienna) validated for yeast by western blot in ref 9

mouse monoclonal anti-GST (1:1,000, 2H3-D10, Monoclonal Antibody Facility, Max Perutz Labs, Vienna), validated by western blot in Eisenhardt et al., Methods Enzymol, doi:10.1016/bs.mie.2018.12.025,

rabbit polyclonal anti-ATG13, (1:50, 5HY-C1-F8, Monoclonal Antibody Facility, Max Perutz Labs, Vienna) validated for U2OS and HEK293 cells by western blot, described and validated in 44

mouse monoclonal anti-RFP (1:1,000, 6g6, Ref No. 6g6-100 Lot No51020014AB-05, Chromotek). validated by the company using transient expression of mRFP, mCherry, mPlum, mOrange, mRFPruby, DsRed, mScarlet and tdTomato on HEK 293T cells by western blot. Ref: Barucci G et al., Nat Cell Biol. 2020, doi:10.1038/s41556-020-0462-7.

Eukaryotic cell lines

Policy information about cell lines and Sex and Gender in Research

Cell line source(s)	HEK293 (R78007, ThermoFisher Scientific), U2OS (K650001, Thermo Fisher Scientific), spodoptera frugiperda Sf9 (94-001F, Biotrend)
Authentication	No authentication has been performed.
Mycoplasma contamination	All of the cell lines used were regularly checked for mycoplasma contamination and were always negative.
Commonly misidentified lines (See ICLAC register)	No commonly misidentified cell lines were used.

Plants

Seed stocks	not used in the study
Novel plant genotypes	not used in the study
Authentication	not used in the study

Flow Cytometry

Plots

Confirm that:

☒ The axis labels state the marker and fluorochrome used (e.g. CD4-FITC).

☒ The axis scales are clearly visible. Include numbers along axes only for bottom left plot of group (a 'group' is an analysis of identical markers).

☒ All plots are contour plots with outliers or pseudocolor plots.

☒ A numerical value for number of cells or percentage (with statistics) is provided.

Methodology

Sample preparation	U2OS cells (cCE377) were seeded in 6-well plates at a density of 500,000 cells per well and cultured in DMEM media supplemented with 10% FBS. After 24 h, the media in the plates were replaced with one containing 1 µg/ml of doxycycline to induce the expression of 2xFKBP-GFP-ULK1. Additionally, in the specified samples, 0.5 µM of rapalog was added for a 24 h incubation period. Before harvesting, the indicated samples were treated with 200 nM Bafilomycin A1 (Cell Signaling Technology, 54645) and rapalog. Following the treatment, cells were washed with DPBS and detached with trypsin-EDTA (Sigma, T3924). The cells were then harvested in FACS medium (phenol-red free DMEM, Sigma, D1145-500ML, supplemented with 10% FBS). Cells were transferred to 1.5 ml tubes and centrifuged at 500 x g for 3 min at room temperature. The supernatant was removed, and the cell pellets were resuspended in 200 µl of FACS media and transferred to U-bottom 96-well plates (Greiner Bio-One, 650970).
Instrument	CytExpert 2.3 analysis software, FlowJo V.10.9.0 - May 5, 2023
Software	CytExpert 2.3 analysis software, FlowJo V.10.9.0 - May 5, 2023
Cell population abundance	at least 50,000 events were analysed out of the initial at least 200,000 events, which accounts for 25% (alive, singlets, and GFP positive)
Gating strategy	The gating strategy is described in detail under https://github.com/hollenstein/sourcecode_mkeima-assay_licheva-et-al-2024

☒ Tick this box to confirm that a figure exemplifying the gating strategy is provided in the Supplementary Information.
