# Supplementary material for: Phase separation of initiation hubs on cargo is a trigger switch for selective autophagy
Source: Nat Cell Biol. 2025 Jan 7;27(2):283–97. doi: 10.1038/s41556-024-01572-y (PMC11821514; doi:10.1038/s41556-024-01572-y)
Supplement: Supplementary file 24 — Unprocessed western blots. [file 41556_2024_1572_MOESM24_ESM.pdf]

1f

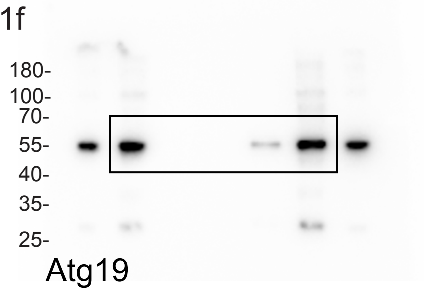

180-
100-
70-
55-
40-
35-
25-
Atg19

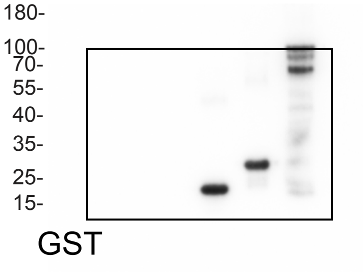

180-
100-
70-
55-
40-
35-
25-
15-
GST

1g

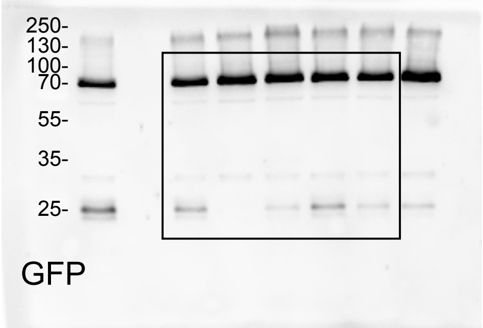

250-
130-
100-
70-
55-
35-
25-
GFP
