# Supplementary figures and images for: Phase separation of initiation hubs on cargo is a trigger switch for selective autophagy
Source: Nat Cell Biol. 2025 Jan 7;27(2):283–97. doi: 10.1038/s41556-024-01572-y (PMC11821514; doi:10.1038/s41556-024-01572-y)

4f

130  
100  
70  
55  
35  
25

GFP

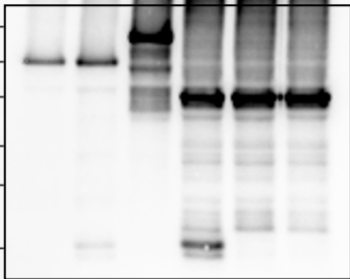

Supplement: Supplementary file 26 — Unprocessed western blots. [file 41556_2024_1572_MOESM26_ESM.pdf]

8e

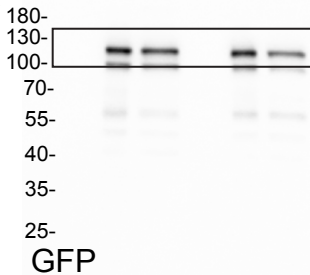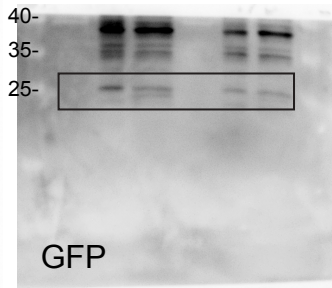

ATG13

130-  
70-  
55-

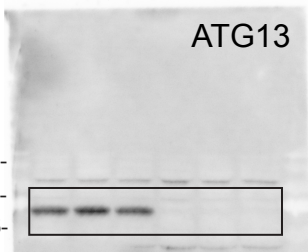

250-

100-

70-

55-

35-

RFP

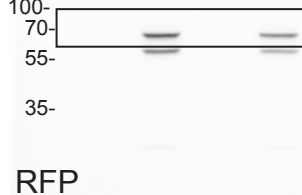

Supplement: Supplementary file 27 — Unprocessed western blots. [file 41556_2024_1572_MOESM27_ESM.pdf]

ED1a

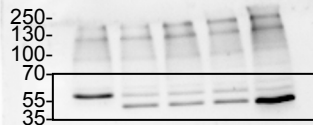

25-  
Ape1

ED1b

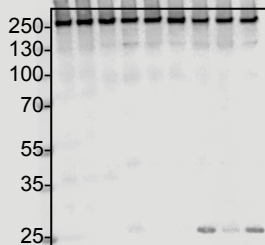

GFP

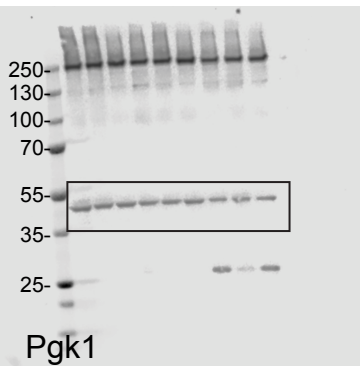

Pgk1

Supplement: Supplementary file 28 — Unprocessed western blots. [file 41556_2024_1572_MOESM28_ESM.pdf]

ED6a

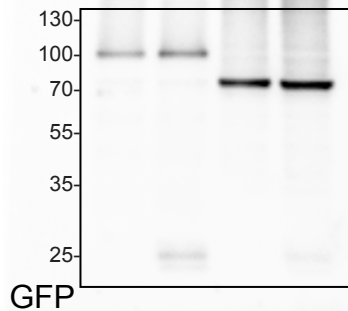

ED6b

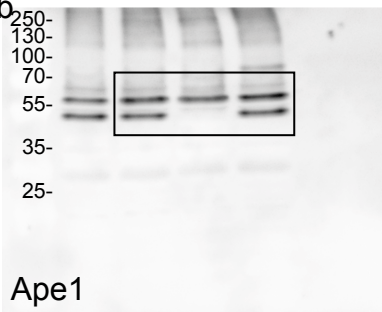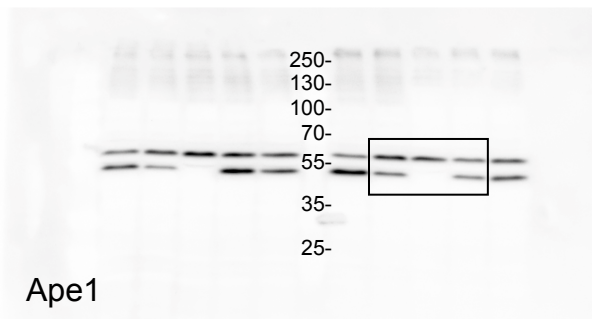

Supplement: Supplementary file 29 — Unprocessed western blots. [file 41556_2024_1572_MOESM29_ESM.pdf]
